# Volunteer participation differentially moderates the association between insomnia and poor subjective well-being in community-dwelling older adults: the Yilan study, Taiwan

**DOI:** 10.1186/s12877-022-03004-8

**Published:** 2022-04-13

**Authors:** Yu-Ting Wang, Nai-Wei Hsu, Yen-Huai Lin, Hsiao-Ting Chang, Pesus Chou, Hsi-Chung Chen

**Affiliations:** 1 Department of Psychiatry, Songde Branch, Taipei City Hospital, Taipei, Taiwan; 2 Department of Internal Medicine & Community Medicine Center, Division of Cardiology, National Yang Ming ChiaoTung University Hospital, Yilan, Taiwan; 3 Faculty of Medicine, School of Medicine, National Yang Ming ChiaoTung University, Taipei, Taiwan; 4 Public Health Bureau, Yilan County, Taiwan; 5 Department of Medical Imaging, Cheng Hsin General Hospital, Taipei, Taiwan; 6 Department of Family Medicine, Taipei Veterans General Hospital, Taipei, Taiwan; 7 Community Medicine Research Center & Institute of Public Health, National Yang Ming ChiaoTung University, Taipei, Taiwan; 8 Department of Psychiatry and Center for Sleep Disorders, National Taiwan University Hospital. No, 7 Chung San South Road, Taipei, 10002 Taiwan

**Keywords:** Insomnia, Volunteer, Subjective well-being, The Yilan Study

## Abstract

**Objectives:**

We aimed to elucidate the moderating effect of volunteer participation on the association between insomnia and subjective well-being.

**Methods:**

This was a community-based, cross-sectional study that targeted community-dwelling older adults aged ≥ 65 years in Yilan city, Taiwan. Whether individuals had volunteered in the past month was asked. Insomnia was measured using the Athens Insomnia Scale-5. Subjective well-being was evaluated using self-rated health, self-rated happiness, the physical component summary (PCS), and the mental component summary (MCS) of Short-form 12. Interaction terms between volunteer participation and insomnia were examined to test the moderating effect of volunteer participation on subjective well-being.

**Results:**

In total, 3,875 participants were included in the study. After controlling for confounders, older adults with insomnia were more likely to have poor subjective well-being, except with respect to PCS. By contrast, volunteering was associated with a low risk of association between self-rated health and happiness. The interaction terms for volunteering with self-rated happiness (*p* = 0.03) and the MCS (*p* = 0.02) were significant. The association between insomnia and poor self-rated happiness among volunteers (odds ratio [OR] = 3.91, 95% confidence interval [CI] = 1.85–8.28) was significantly stronger than that in non-volunteers (OR = 1.48, 95% CI = 1.18–1.86). However, insomnia was linked with poor MCS in non-volunteers (OR = 1.53, 95% CI = 1.21–1.94), but not in volunteers (OR = 0.64, 95% CI = 0.27–1.50).

**Discussion:**

Volunteer participation moderated the association between insomnia and subjective well-being; specifically, volunteering strengthened the association between insomnia and poor self-rated happiness but mitigated the relationship between insomnia and poor MCS.

**Supplementary Information:**

The online version contains supplementary material available at 10.1186/s12877-022-03004-8.

## Introduction

Insomnia is a prevalent health problem [1] associated with reduced happiness [2], higher risk of mental illnesses [3], and a poor quality of life [4]. Older adults are more susceptible to insomnia [5] because of age-related changes in their sleep patterns, increased morbidities, and psychosocial stressors [6]. Insomnia in older adults is correlated specifically with physical disabilities [7], falls [6], mortality [6], depression [8], lower life satisfaction [9], and a poor quality of life [10]; thus, reducing the impact of insomnia among older adults is important in geriatric medicine.

A previous study found that increased social participation among older adults was related to decreased insomnia [11]. Considering social participation, volunteer participation was especially promoted in the old age group as it improves the health of participants and benefits the community [12]. There is abundant literature supporting that volunteer participation is generally beneficial for mental and physical health [13–19]. In the general population, volunteers were happier with a higher life satisfaction [16] and better self-rated health [15]. The advantage of volunteer participation was more prominent in older adults than among younger adults [17]. Compared to younger volunteers, older volunteers had fewer physical limitations [18], lower mortality risks [19], and lesser episodes of depression [17]. Further, volunteering in older adults is related to subjective well-being. Subjective well-being is defined as one’s appraisal to its affective well-being (i.e. the presence of positive feelings and absence of negative feelings) and cognitive well-being (i.e. the evaluation of one’s life in overall and specific domains) [20]. Although literature revealed that older volunteers may have better subjective well-being, including higher positive affect [21], better self-rated health, and happiness [14], volunteering seemed to have differential impact on various domains of subjective well-being. For instance, comparing volunteer participation with other productive engagement (e.g. taking care of grandchildren and being involved in educational activities or religious groups), volunteer participation in older adults was not significantly related to better self-rated health [22]. Volunteering also failed to predict better quality of life [23]. Interestingly, volunteering exerted contrasting influences on different domains of subjective well-being. Specifically, volunteering benefited subjective health and life satisfaction but may also correlate with higher depressive symptoms in an older population [24]. Therefore, although volunteer participation is often promoted worldwide to enhance both physical and mental health, it remains equivocal whether volunteer participation is beneficial for subjective well-being in older adults.

Physiological aging impairs physical health; therefore, identifying factors that can alleviate the adverse impact of insomnia and maintain subjective well-being is imperative to achieving successful aging [25]. Thus, the extent to which volunteer participation by older adults influences the impact of insomnia on subjective well-being is particularly relevant in an aging society. Theoretically, the personality traits of an altruistic attitude [26], low neuroticism, and high extraversion of volunteers related to better self-rated health [27] may neutralize the adverse impact of insomnia on subjective well-being. In contrast, other characteristics of volunteers, such as conscientiousness [28], may exaggerate the unfavorable influence of insomnia on subjective well-being because they may conflict with the core nature of good sleep, including automaticity (circadian and homeostatic regulation) and plasticity (accommodation to real-world circumstances) [29]. In the literature, only one study has demonstrated how ethnicity moderates the association between insomnia and positive mood [30], one domain of subjective well-being. No studies have examined the influence of volunteer participation on the relationship between insomnia and comprehensive domains of subjective well-being in older adults.

Therefore, we conducted this study with two objectives. First, relationships between insomnia and subjective well-being as well as volunteer participation and subjective well-being were examined in community dwelling older adults. Second, the moderating effect of volunteer participation on the association between insomnia and poor subjective well-being was explored. We hypothesized the following: (1) Insomnia is related to poor subjective well-being while volunteer participation is related to better subjective well-being, and (2) the status of volunteer participation modifies the magnitude of association between insomnia and poor subjective well-being.

## Methods

### Study design and participants

This was a community-based, cross-sectional study that was under “the Yilan study” [31]. Data were collected from 2013 to 2016. Community-dwelling older adults (aged ≥ 65 years) were randomly recruited and interviewed by trained research assistants. The anthropometric data were collected by physical examinations, while information on sociodemographic characteristics, lifestyle, medical history, and subjective well-being was collected through face-to-face interviews. Participants who were incapable of completing interviews or who could not respond to questions owing to diseases or any other causes were excluded. This study was approved by the institutional review board of National Yang-Ming University Hospital (Intuitional Review Board number: 2011A016); it followed the principles of Declaration of Helsinki. All the participants provided written informed consent.

### Measurement of sociodemographics, lifestyle, and body mass index

All participants were categorized into those aged ≥ 75 and < 75 years, and thus older adults were classified as “old-old” and “young-old,” respectively [32]. Detailed academic achievements were also obtained. Except for illiterate participants, all others were collapsed into one group as literate. Marital status was classified as married, single/divorced/separated, or widowed. Living status was grouped as living with others or living alone. Frequency of exercise of ≥ 3 per week was defined as “regular exercise.” Information regarding current substance use, including cigarettes and alcohol, was also collected. Use status was categorized as non-user, ex-user, or current user. Body mass index was separated into four categories: < 18.5 kg/m^2^ as underweight, 18.5–23.9 kg/m^2^ as appropriate, > 23.9 kg/m^2^ as overweight, and those who could not be assessed because they had physical diseases or were bed-ridden (these individuals were categorized as disabled) [33].

### Measurement and definition for poor subjective well-being

Subjective well-being generally included affective well-being and cognitive well-being [34]; thus, we considered “self-rated happiness” as positive affective well-being and “the mental component summary (MCS) of the Short-Form 12 Health Survey Version 2 (SF-12v2) as negative affective well-being. Meanwhile, “the physical component summary (PCS) of the SF-12v2” and “self-rated health” corresponded to specific domains of cognitive well-being. Two global questions, i.e., “How would you rate your present health status?” and “In general, how would you rate your current state of happiness?” were used to evaluate self-rated health and happiness, respectively. Participants were assigned scores from 0 to 100 for these two questions. The Chinese version of the global questions for self-rated health and self-rated happiness was validated previously [35]. Specifically, the convergent validity of self-rated health with PCS is 0.471 (*p* < 0.001); it’s divergent validity with Groningen Activity Restriction Scale is -0.316 (*p* < 0.001); the convergent validity of self-rated happiness with MCS is 0.357 (*p* < 0.001), and it’s divergent validity with Hospital Anxiety and Depression Scale is -0.423 (*p* < 0.001) [35]. The SF-12v2 was used to evaluate the health-related quality of life (HRQoL) in the past four weeks. A higher score indicated a better HRQoL [36]. The Chinese version of SF-12 was also validated previously [37]. We defined “poor subjective well-being” as the lowest tertile in each measurement of subjective well-being because a non-linear relationship was observed [38]. Meanwhile, to prevent a double negative statement, those with less likelihood of lowest tertile in each measurement of subjective well-being were designated “better subjective well-being” throughout the manuscript.

### Volunteer participation

Volunteer experience was determined by asking the following question to each participant: “In the past month, did you participate in any kind of volunteer service in the community?” A positive response implied volunteer participation.

### Measurement of insomnia

The five-item version of the Athens Insomnia Scale was used to evaluate insomnia [39]. The Chinese version of this scale had satisfactory reliability and validity [40]. When the cutoff points were five and above, the sensitivity and specificity for identifying insomnia were 88.2% and 78.3%, respectively, and the area under the receiver-operating characteristic curve was 0.90. We did not formally diagnose insomnia; thus, a score ≥ 5 was considered to indicate “insomnia with clinical significance.”

### Other covariates

#### Other sleep–wake related variables

Snoring was used to evaluate breathing disorders during sleep. A history of hypnotic use was recorded by asking the following question: “In the past month, have you used any sleep-inducing drugs?” Daytime sleepiness was evaluated using the Epworth Sleepiness Scale [41]. The Chinese version of the Epworth Sleepiness Scale was proven to have adequate validity [42]. Individuals with excessive daytime sleepiness had a total score ≥ 11 [41].

#### Physical comorbidities

Physical comorbidities were identified by participants’ self-reports of certain diseases and their corresponding treatments, including diabetes mellitus, hypertension, heart disease, hyperlipidemia, and stroke. Physical restriction was defined using the Groningen Activity Restriction Scale, which comprises 18 daily activities [43]. The older adults who participated in this study were usually active and functioning in the community. If more than one daily activity was performed with difficulty (total score ≥ 19), the participant was considered to have physical restrictions.

#### Evaluation of anxiety and depression

The Hospital Anxiety and Depression Scale was used to evaluate symptoms of anxiety and depression. This scale is a valid instrument for evaluating clinical and subclinical depression and anxiety in the general population [44]. Older adults often have various morbidities; thus, when symptoms correspond to the somatic domain of depressive and anxiety symptomatology, the Hospital Anxiety and Depression Scale asks questions related to the non-physical symptoms of depression and anxiety. It consists of 14 items that evaluate individuals’ symptoms in the past week: seven items measure anxiety and seven measure depression. All items are scored from 0 to 3. Higher scores indicate more severe anxiety and depression. This scale was validated in older Chinese adults; the cutoffs were 3 and 6 points for anxiety and depression, respectively [45].

### Statistical analysis

The goodness of fit test was used to compare demographic differences between the study participants and the registered individuals in Yilan city. Considering the first aim of this study, Chi-square test was used to explore the bivariate relationship between variables and subjective well-being. To control for confounding effects, four individual multiple logistic regression analyses were used to examine the independent relationship between insomnia and volunteer participation with each measurement of poor subjective well-being. All variables included in the bivariate analyses were accommodated in multivariable logistic regression analyses as covariates. Considering the second aim of this study, the interaction term of volunteer participation and Athens Insomnia Scales (volunteer participation x insomnia) was examined by four individual multiple logistic regression models to examine whether volunteer participation moderated the associations between insomnia and poor subjective well-being. If the interaction terms were statistically significant, stratified analyses by volunteer participation were performed to illustrate the differential magnitude of associations between insomnia and poor subjective well-being. Additionally, sensitivity analyses were also performed to examine the variation of the significance of interaction terms by different cutoffs for the poor subjective well-being. Herein, data were missing completely at random; thus, the missing values were omitted, and the method of available-case analysis was used. *P-*value less than 0.05 was considered statistically significant. Statistical analysis was conducted using SPSS for Windows, version 17.0 (SPSS Inc., Chicago, IL, USA).

## Results

In total, 3,785 participants were included in the study. Over half of the respondents were aged ≥ 75 years (56.2%); the average age was 76.4 ± 6.6 years and females comprised 56.9% of the participants. One in five participants (21.3%) had Athens Insomnia Scale-defined insomnia, and one in nine (11.4%) volunteered in the past month (Table 1). The participants in this study were older (*χ*^2^ = 4.11, df = 1, *p* = 0.04) with similar sex distribution (*χ*^2 ^= 0.52, df = 1,* p* = 0.47; they were also more illiterate (*χ*^2 ^=14.58, df = 1, *p* < 0.001) compared to the demographic data provided by the Yilan household registration offices in 2013 [46] (Supplementary table S1). The bivariate associations among sociodemographic factors, lifestyle factors, and clinical characteristics with the four measurements of subjective well-being are demonstrated in Supplementary table S2 and S3. Thus, volunteer participation was associated with better self-rated health (*p* < 0.001), self-rated happiness (*p* < 0.001), PCS (*p* < 0.001), and MCS (*p* = 0.01) (Supplementary table S2). Depression (all *p* < 0.001), anxiety (all *p* < 0.001), physical disabilities (all *p* < 0.001), insomnia (all *p* < 0.001), and use of hypnotics in the past month (all *p* < 0.001) were significantly related to all dimensions of poor subjective well-being (Supplementary table S3).

**Table 1:** Sociodemographic and clinical characteristics of participants (*n* = 3785)^*^

	n (%)
Age (years)	
< 75	1656 (43.8)
≥ 75	2129 (56.2)
Sex	
Female	2155 (56.9)
Body mass index (kg/m^2^)	
< 18.5	159 (4.2)
18.5–23.9	1440 (38.1)
> 23.9	2007 (53.0)
Disabled	178 (4.7)
Education status	
Literate	3003 (79.3)
Illiterate	782 (20.7)
Marital status	
Married	2465 (65.3)
Single/ divorced/ separated	56 (1.5)
Widowed	1252 (33.2)
Living status	
With others	3358 (88.8)
Alone	425 (11.2)
Frequency of exercise per week ≥ 3/ week	2193 (58.0)
Smoking status	
Non-smoker	2866 (75.7)
Ex- smoker	568 (15.0)
Current smoker	351 (9.3)
Drinking status	
Non-drinker	3094 (81.7)
Ex- drinker	198 (5.2)
Current drinker	493 (13.0)
Medical history	
Diabetes mellitus	888 (23.5)
Hypertension	2152 (56.9)
Heart disease	1197 (31.7)
Hyperlipidemia	841 (22.3)
Stroke	194 (5.1)
Snore	43 (1.2)
Hospital Anxiety Depression Scale	
Depression ≥ 6	443 (11.7)
Anxiety ≥ 3	1345 (35.5)
Groningen Activity Restriction Scale ≥ 19	1159 (44.2)
Taking hypnotics in the past one month	849 (22.5)
Epworth Sleepiness Scale ≥ 11	602 (15.9)
Athens Insomnia Scale ≥ 5	806 (21.3)
Volunteer participation	428 (11.4)

^*^Numbers of participants do not equal 3785 because of missing values in variables

Table 2 shows multiple logistic regression results for factors associated with subjective well-being. After controlling for various covariates, insomnia indicated a high likelihood of poor self-rated health (Odds Ratio [OR] = 1.58, 95% confidence interval [CI] = 1.27–1.96, *p* < 0.001), self-rated happiness (OR = 1.58, 95% CI = 1.26–1.97, *p* < 0.001), and the MCS (OR = 1.42, 95% CI = 1.14–1.78, *p* = 0.002), and its association with poor PCS was of borderline significance (OR = 1.30, 95% CI = 0.99–1.72, *p* = 0.06). By contrast, volunteer participation was associated with better self-rated health (OR = 0.66, 95% CI = 0.49–0.88, *p* = 0.01) and self-rated happiness (OR = 0.56, 95% CI = 0.42–0.74,* p* < 0.001); however, the associations with better PCS and MCS in the bivariate analysis disappeared.

**Table 2:** Multiple logistic regression analyses for factors associated with unfavorable subjective well-being

	Self-rated				Short Form-12			
	Health		Happiness		Physical component summary		Mental component summary	
	AOR (95% CI)	*p*-value	AOR (95% CI)	*p*-value	AOR (95% CI)	*p*-value	AOR (95% CI)	*p*-value
Age (≥ 75 vs. < 75 years)	0.77 (0.63–0.93)	0.01	0.90 (0.75–1.09)	0.28	1.24 (0.96–1.61)	0.10	0.94 (0.77–1.16)	0.58
Sex (male vs. female)	1.17 (0.90–1.52)	0.24	1.31 (1.02–1.68)	0.04	1.26 (0.89–1.79)	0.20	1.37 (1.04–1.80)	0.03
Body mass index (kg/m^2^)								
< 18.5 vs. 18.5–23.9	1.53 (0.97–2.43)	0.07	1.25 (0.79–1.97)	0.35	1.42 (0.76–2.63)	0.27	1.25 (0.77–2.04)	0.37
> 23.9 vs. 18.5–23.9	0.96 (0.79–1.16)	0.68	0.93 (0.78–1.12)	0.46	0.96 (0.74–1.23)	0.74	0.86 (0.70–1.06)	0.15
Disabled vs. 18.5–23.9	1.91 (1.23–2.95)	0.004	1.20 (0.77–1.87)	0.43	7.15 (3.45–14.83)	< 0.001	2.16 (1.39–3.34)	0.001
Education status (illiterate vs. literate)	1.46 (1.17–1.82)	0.001	1.74 (1.40–2.16)	< 0.001	1.80 (1.36–2.40)	< 0.001	0.88 (0.69–1.11)	0.27
Marital status								
Single/ divorced/ separated vs. Married	1.60 (0.78–3.30)	0.20	1.63 (0.76–3.49)	0.21	1.72 (0.71–4.18)	0.23	1.07 (0.49–2.32)	0.86
Widowed vs. Married	0.96 (0.78–1.19)	0.72	1.26 (1.03–1.54)	0.03	0.85 (0.64–1.12)	0.24	0.99 (0.79–1.24)	0.91
Living status (alone vs. with others)	1.00 (0.74–1.36)	0.98	1.04 (0.78–1.39)	0.78	1.31 (0.88–1.94)	0.18	1.00 (0.73–1.38)	0.98
Frequency of exercise per week (≥ 3 vs. < 3 / week)	1.21 (1.00–1.45)	0.045	1.32 (1.11–1.58)	0.002	1.52 (1.20–1.92)	0.001	1.12 (0.92–1.36)	0.27
Volunteer (yes vs. no)	0.66 (0.49–0.88)	0.01	0.56 (0.42–0.74)	< 0.001	0.71 (0.47–1.06)	0.09	1.03 (0.76–1.40)	0.83
Smoking status								
Ex- smoker vs. Non-smoker	1.26 (0.93–1.72)	0.14	1.43 (1.06–1.92)	0.02	1.07 (0.71–1.61)	0.76	0.79 (0.57–1.11)	0.18
Current smoker vs. Non-smoker	0.91 (0.63–1.31)	0.61	1.36 (0.97–1.90)	0.08	0.90 (0.55–1.48)	0.67	0.81 (0.55–1.20)	0.30
Drinking status								
Ex- drinker vs. Non-drinker	0.99 (0.65–1.50)	0.95	1.01 (0.67–1.52)	0.97	1.10 (0.63–1.91)	0.74	0.39 (0.23–0.66)	< 0.001
Current drinker vs. Non-drinker	0.85 (0.64–1.14)	0.28	0.74 (0.56–0.98)	0.04	0.65 (0.44–0.96)	0.03	0.63 (0.45–0.87)	0.01
Medical history								
Diabetes mellitus (yes vs. no)	1.51 (1.22–1.85)	< 0.001	1.00 (0.81–1.22)	0.97	1.51 (1.15–2.00)	0.004	1.21 (0.96–1.51)	0.10
Hypertension (yes vs. no)	1.30 (1.08–1.58)	0.01	1.25 (1.04–1.50)	0.02	1.11 (0.86–1.43)	0.44	0.96 (0.78–1.18)	0.68
Heart disease (yes vs. no)	1.26 (1.04–1.53)	0.02	1.00 (0.83–1.21)	0.99	1.49 (1.16–1.91)	0.002	0.86 (0.69–1.06)	0.15
Hyperlipidemia (yes vs. no)	1.15 (0.93–1.43)	0.20	1.15 (0.93–1.43)	0.19	0.95 (0.71–1.27)	0.73	0.94 (0.74–1.19)	0.60
Stroke (yes vs. no)	1.15 (0.76–1.76)	0.50	1.01 (0.66–1.55)	0.96	2.76 (1.53–4.98)	0.001	1.13 (0.73–1.76)	0.59
Snore (yes vs. no)	1.55 (0.69–3.50)	0.29	0.82 (0.37–1.85)	0.64	3.62 (1.26–10.42)	0.02	0.52 (0.17–1.56)	0.24
Hospital Anxiety Depression Scale								
Depression (≥ 6 vs. < 6)	1.49 (1.08–2.05)	0.02	2.28 (1.58–3.30)	< 0.001	1.11 (0.75–1.66)	0.60	5.74 (3.98–8.29)	< 0.001
Anxiety (≥ 3 vs. < 3)	1.78 (1.46–2.16)	< 0.001	2.16 (1.78–2.62)	< 0.001	1.16 (0.90–1.51)	0.26	3.25 (2.66–3.97)	< 0.001
Groningen Activity Restriction Scale (≥ 19 vs. < 19)	2.59 (2.15–3.13)	< 0.001	1.89 (1.57–2.27)	< 0.001	27.99 (21.41–36.59)	< 0.001	1.66 (1.35–2.03)	< 0.001
Taking hypnotics in the past one month (yes vs. no)	1.69 (1.38–2.09)	< 0.001	1.56 (1.26–1.92)	< 0.001	1.34 (1.02–1.76)	0.04	1.78 (1.43–2.21)	< 0.001
Epworth Sleepiness Scale (≥ 11 vs. < 11)	1.14 (0.87–1.50)	0.34	0.99 (0.75–1.30)	0.95	1.11 (0.78–1.57)	0.57	1.93 (1.45–2.56)	< 0.001
**Athens Insomnia Scale (≥ 5 vs. < 5)**	1.58 (1.27–1.96)	< 0.001	1.58 (1.26–1.97)	< 0.001	1.30 (0.99–1.72)	0.06	1.42 (1.14–1.78)	0.002
**Volunteer (yes vs. no)**	0.66 (0.49–0.88)	0.01	0.56 (0.42–0.74)	< 0.001	0.71 (0.47–1.06)	0.09	1.03 (0.76–1.40)	0.83

*AOR* adjusted odds ratio

We further examined moderation effects of volunteer participation on the association between insomnia and subjective well-being by testing the interaction terms (Volunteer participation × Athens Insomnia Scale) (Table 3). Volunteer participation moderated the relationships of insomnia with self-rated happiness (*p* = 0.03) and MCS (*p* = 0.02); it did not affect the relationships of insomnia with self-rated heath (*p* = 0.83) and PCS (*p* = 0.61). Thus, stratified analyses by volunteering were performed to compare the magnitude of associations between insomnia and these two measurements of subjective well-being (Table 4). When they were stratified further, the associations of insomnia with self-rated happiness and MCS by status of volunteer participation revealed a stronger association of insomnia with poor self-rated happiness in volunteers (OR = 3.91, 95% CI = 1.85–8.28, *p* < 0.001) compared to non-volunteers (OR = 1.48, 95% CI = 1.18–1.86, *p* = 0.001). By contrast, insomnia was not associated with poor MCS in older adults with recent volunteering experience (OR = 0.64, 95% CI = 0.27–1.50, *p* = 0.30); however, insomnia was associated with poor MCS in non-volunteers (OR = 1.53, 95% CI = 1.21–1.94, *p* < 0.001).

**Table 3:** Multiple logistic regression analyses to examine the interaction effects of volunteering and insomnia on unfavorable subjective well-being

	Self-rated				Short Form-12			
	Health		Happiness		Physical component summary		Mental component summary	
	AOR (95% CI)	*p*-value	AOR (95% CI)	*p*-value	AOR (95% CI)	*p*-value	AOR (95% CI)	*p*-value
Age (≥ 75 vs. < 75 years)	0.77 (0.63–0.93)	0.01	0.90 (0.75–1.08)	0.27	1.25 (0.96–1.61)	0.10	0.94 (0.77–1.16)	0.58
Sex (male vs. female)	1.17 (0.90–1.52)	0.24	1.31 (1.02–1.69)	0.03	1.26 (0.89–1.79)	0.20	1.37 (1.03–1.80)	0.03
Body mass index (kg/m^2^)								
< 18.5 vs. 18.5–23.9	1.53 (0.96–2.42)	0.08	1.23 (0.78–1.95)	0.38	1.41 (0.76–2.61)	0.28	1.27 (0.78–2.07)	0.34
> 23.9 vs. 18.5–23.9	0.96 (0.79–1.16)	0.68	0.93 (0.78–1.12)	0.45	0.96 (0.75–1.23)	0.74	0.86 (0.70–1.06)	0.16
Disabled vs. 18.5–23.9	1.91 (1.23–2.95)	0.004	1.19 (0.77–1.87)	0.44	7.16 (3.45–14.88)	< 0.001	2.16 (1.39–3.34)	0.001
Education status (illiterate vs. literate)	1.46 (1.18–1.82)	0.001	1.75 (1.41–2.18)	< 0.001	1.81 (1.36–2.40)	< 0.001	0.87 (0.68–1.10)	0.24
Marital status								
Single/ divorced/ separated vs. Married	1.61 (0.78–3.30)	0.20	1.66 (0.78–3.55)	0.19	1.73 (0.71–4.21)	0.23	1.05 (0.49–2.29)	0.90
Widowed vs. Married	0.96 (0.78–1.19)	0.72	1.27 (1.03–1.55)	0.02	0.85 (0.64–1.12)	0.24	0.99 (0.79–1.24)	0.91
Living status (alone vs. with others)	1.00 (0.74–1.36)	0.98	1.05 (0.79–1.40)	0.75	1.31 (0.88–1.93)	0.18	1.00 (0.73–1.38)	0.98
Frequency of exercise per week (≥ 3 vs. < 3 / week)	1.21 (1.00–1.45)	0.046	1.32 (1.11–1.58)	0.002	1.51 (1.19–1.92)	0.001	1.12 (0.92–1.36)	0.25
Smoking status								
Ex- smoker vs. Non-smoker	1.26 (0.93–1.72)	0.14	1.42 (1.06–1.91)	0.02	1.06 (0.70–1.61)	0.77	0.80 (0.57–1.12)	0.19
Current smoker vs. Non-smoker	0.91 (0.63–1.31)	0.61	1.35 (0.96–1.90)	0.08	0.90 (0.55–1.48)	0.68	0.81 (0.55–1.20)	0.29
Drinking status								
Ex- drinker vs. Non-drinker	0.99 (0.65–1.50)	0.94	1.00 (0.67–1.51)	0.99	1.10 (0.63–1.92)	0.74	0.40 (0.24–0.66)	< 0.001
Current drinker vs. Non-drinker	0.85 (0.64–1.14)	0.28	0.75 (0.57–0.99)	0.04	0.65 (0.44–0.96)	0.03	0.62 (0.45–0.86)	0.004
Medical history								
Diabetes mellitus (yes vs. no)	1.51 (1.22–1.86)	< 0.001	1.00 (0.81–1.23)	0.99	1.52 (1.15–2.01)	0.003	1.20 (0.96–1.51)	0.11
Hypertension (yes vs. no)	1.30 (1.08–1.58)	0.01	1.26 (1.04–1.51)	0.02	1.11 (0.86–1.43)	0.42	0.95 (0.77–1.16)	0.60
Cardiovascular disease (yes vs. no)	1.26 (1.04–1.53)	0.02	1.00 (0.83–1.21)	0.99	1.48 (1.15–1.91)	0.002	0.86 (0.70–1.06)	0.16
Hyperlipidemia (yes vs. no)	1.15 (0.93–1.43)	0.20	1.15 (0.93–1.42)	0.021	0.95 (0.71–1.26)	0.70	0.95 (0.75–1.20)	0.65
Stroke (yes vs. no)	1.15 (0.76–1.76)	0.50	1.01 (0.66–1.54)	0.97	2.76 (1.53–4.98)	0.001	1.14 (0.73–1.77)	0.57
Snore (yes vs. no)	1.55 (0.69–3.49)	0.30	0.80 (0.35–1.79)	0.58	3.57 (1.24–10.28)	0.02	0.54 (0.18–1.64)	0.28
Hospital Anxiety Depression Scale								
Depression (≥ 6 vs. < 6)	1.49 (1.08–2.05)	0.02	2.29 (1.58–3.30)	< 0.001	1.12 (0.75–1.66)	0.59	5.77 (3.99–8.34)	< 0.001
Anxiety (≥ 3 vs. < 3)	1.78 (1.46–2.16)	< 0.001	2.17 (1.79–2.64)	< 0.001	1.16 (0.90–1.51)	0.26	3.24 (2.66–3.96)	< 0.001
Groningen Activity Restriction Scale (≥ 19 vs. < 19)	2.59 (2.15–3.13)	< 0.001	1.89 (1.57–2.27)	< 0.001	27.98 (21.40–36.58)	< 0.001	1.66 (1.35–2.03)	< 0.001
Taking hypnotics in the past one month (yes vs no)	1.69 (1.38–2.09)	< 0.001	1.55 (1.26–1.92)	< 0.001	1.34 (1.02–1.76)	0.04	1.78 (1.43–2.22)	< 0.001
Epworth Sleepiness Scale (≥ 11 vs. < 11)	1.14 (0.87–1.50)	0.35	0.98 (0.75–1.29)	0.90	1.10 (0.78–1.56)	0.60	1.95 (1.47–2.60)	< 0.001
**Athens Insomnia Scale (≥ 5 vs. < 5)**	1.57 (1.25–1.96)	< 0.001	1.46 (1.16–1.84)	0.001	1.28 (0.96–1.71)	0.10	1.55 (1.22–1.96)	< 0.001
**Volunteer (yes vs. no)**	0.65 (0.47–0.90)	0.01	0.48 (0.35–0.66)	< 0.001	0.67 (0.42–1.06)	0.09	1.22 (0.88–1.70)	0.23
**Athens Insomnia Scale x Volunteer**	1.09 (0.52–2.25)	0.83	2.26 (1.10–4.65)	0.03	1.29 (0.49–3.44)	0.61	0.39 (0.18–0.87)	0.02

*AOR* adjusted odds ratio

**Table 4:** Multiple logistic regression analyses for factors associated with unfavorable self-rated happiness and mental component summary stratified by volunteer participation

	Self-rated Happiness				Short Form-12 Mental component summary			
	Volunteer				Volunteer			
	Yes		No		Yes		No	
	AOR (95% CI)	*p*-value	AOR (95% CI)	*p*-value	AOR (95% CI)	*p*-value	AOR (95% CI)	*p*-value
Age (≥ 75 vs. < 75 years)	0.70 (0.38–1.28)	0.25	0.93 (0.76–1.13)	0.47	1.12 (0.59–2.11)	0.73	0.91 (0.73–1.13)	0.40
Sex (male vs. female)	1.51 (0.67–3.41)	0.32	1.28 (0.98–1.66)	0.07	2.28 (0.98–5.30)	0.06	1.29 (0.95–1.73)	0.10
Body mass index (kg/m^2^)								
< 18.5 vs. 18.5–23.9	1.40 (0.22–8.95)	0.72	1.22 (0.76–1.96)	0.42	0.84 (0.14–4.94)	0.84	1.22 (0.73–2.03)	0.46
> 23.9 vs. 18.5–23.9	0.55 (0.31–1.00)	0.049	0.98 (0.81–1.19)	0.85	0.74 (0.39–1.40)	0.36	0.87 (0.70–1.09)	0.22
Disabled vs. 18.5–23.9	NA^a^	0.99	1.28 (0.81–2.02)	0.29	NA^a^	0.99	2.19 (1.40–3.43)	0.001
Education status (illiterate vs. literate)	1.31 (0.55–3.11)	0.55	1.77 (1.41–2.23)	< 0.001	0.43 (0.15–1.20)	0.11	0.91 (0.71–1.16)	0.45
Marital status								
Single/ divorced/ separated vs. Married	10.16 (0.68–15.58)	0.09	1.40 (0.64–3.06)	0.40	0.41 (0.01–13.97)	0.62	1.11 (0.50–2.47)	0.81
Widowed vs. Married	1.80 (0.89–3.67)	0.40	1.21 (0.98–1.50)	0.08	0.97 (0.44–2.10)	0.93	0.98 (0.78–1.25)	0.89
Living status (alone vs. with others)	1.19 (0.51–2.76)	0.69	1.04 (0.77–1.42)	0.79	0.69 (0.28–1.69)	0.41	1.06 (0.75–1.51)	0.73
Frequency of exercise per week (≥ 3 vs. < 3 / week)	1.26 (0.66–2.43)	0.49	1.33 (1.10–1.60)	0.003	0.88 (0.42–1.84)	0.73	1.16 (0.94–1.43)	0.16
Smoking status								
Ex- smoker vs. Non-smoker	0.83 (0.32–2.17)	0.71	1.55 (1.13–2.13)	0.01	0.26 (0.09–0.75)	0.01	0.91 (0.63–1.31)	0.62
Current smoker vs. Non-smoker	1.35 (0.45–1.02)	0.59	1.38 (0.96–1.97)	0.08	0.29 (0.08–1.07)	0.06	0.92 (0.60–1.39)	0.68
Drinking status								
Ex- drinker vs. Non-drinker	0.78 (0.19–3.27)	0.74	1.02 (0.66–1.58)	0.94	0.28 (0.04–2.01)	0.21	0.39 (0.22–0.67)	0.001
Current drinker vs. Non-drinker	0.89 (0.40–1.98)	0.78	0.72 (0.54–0.97)	0.03	0.66 (0.27–1.59)	0.35	0.59 (0.42–0.85)	0.004
Medical history								
Diabetes mellitus (yes vs. no)	1.02 (0.49–2.08)	0.97	0.98 (0.79–1.21)	0.83	1.78 (0.83–3.82)	0.14	1.16 (0.91–1.48)	0.22
Hypertension (yes vs. no)	1.97 (1.03–3.76)	0.04	1.19 (0.98–1.45)	0.08	0.80 (0.41–1.55)	0.50	0.95 (0.77–1.19)	0.68
Cardiovascular disease (yes vs. no)	1.14 (0.61–2.16)	0.68	0.98 (0.80–1.20)	0.85	0.39 (0.19–0.82)	0.01	0.94 (0.75–1.17)	0.56
Hyperlipidemia (yes vs. no)	0.56 (0.28–1.14)	0.11	1.22 (0.97–1.54)	0.09	0.94 (0.45–1.93)	0.86	0.94 (0.72–1.21)	0.60
Stroke (yes vs. no)	0.45 (0.02–11.25)	0.63	1.01 (0.66–1.56)	0.96	NA^a^	0.99	1.16 (0.74–1.82)	0.52
Snore (yes vs. no)	NA^a^	0.99	0.98 (0.72–2.30)	0.96	2.18 (0.19–24.94)	0.53	0.43 (0.12–1.50)	0.18
Hospital Anxiety Depression Scale								
Depression (≥ 6 vs. < 6)	1.10 (0.27–4.52)	0.89	2.35 (1.60–3.46)	< 0.001	5.74 (1.04–31.73)	0.045	5.86 (4.01–8.58)	< 0.001
Anxiety (≥ 3 vs. < 3)	2.26 (1.21–4.24)	0.01	2.21 (1.80–2.72)	< 0.001	4.14 (2.16–7.96)	< 0.001	3.22 (2.60–3.98)	< 0.001
Groningen Activity Restriction Scale (≥ 19 vs. < 19)	2.11 (1.13–3.92)	0.02	1.86 (1.53–2.26)	< 0.001	1.83 (0.93–3.60)	0.08	1.63 (1.31–2.02)	< 0.001
Taking hypnotics in the past one month (yes vs. no)	2.46 (1.25–4.85)	0.01	1.48 (1.19–1.86)	0.001	1.88 (0.91–3.91)	0.09	1.79 (1.42–2.25)	< 0.001
Epworth Sleepiness Scale (≥ 11 vs. < 11)	0.89 (0.34–2.31)	0.81	0.98 (0.73–1.31)	0.90	2.80 (1.10–7.16)	0.03	1.88 (1.39–2.54)	< 0.001
**Athens Insomnia Scale (≥ 5 vs. < 5)**	3.91 (1.85–8.28)	< 0.001	1.48 (1.18–1.86)	0.001	0.64 (0.27–1.50)	0.30	1.53 (1.21–1.94)	< 0.001

^a^Odds ratios and their 95% confidence intervals were not available due to sparse cases

*AOR* adjusted odds ratio

To examine the influence of different cutoffs for the definition of poor subjective well-being on the moderation effect of volunteer participation, additional sensitivity analyses using median, lowest tertile, and lowest quartile were performed (supplement table S4). Although the direction of moderation effect remained similar across different cutoffs, those with lower medians for each measurement of subjective well-being did not show any statistically significant interaction terms. By contrast, the cutoff with the lowest quartiles only illustrated the moderation effect of volunteering on the association between insomnia and MCS. Thus, the cutoff with median compromised the validity of poor subjective well-being despite having the largest statistical power among three different cutoffs. Meanwhile, the cutoff with the lowest quartile preserved the validity of poor subjective well-being, but it did not have statistical power. Therefore, the sensitivity analyses concluded that the lowest tertile should be the most optimal cutoff to define poor subjective well-being regarding the balance between validity of poor subjective well-being and statistical power in this study.

## Discussion

We used a large sample size, comprehensive measurements of subjective well-being, and various covariates to examine the moderating effect of volunteer participation on the association between insomnia and subjective well-being. The main finding was consistent with our first hypothesis that insomnia and volunteer participation had opposing relationships with subjective well-being. Participants with insomnia had a high likelihood of poor subjective well-being. However, volunteer participation was associated with good subjective well-being. Furthermore, volunteer participation was the effect modifier of the relationship between insomnia and subjective well-being. Although this finding was consistent with our second hypothesis, volunteering unexpectedly had a contrasting effect on the association between insomnia and subjective well-being. Specifically, volunteers with insomnia were more likely to have better MCS compared to non-volunteers. By contrast, volunteers with insomnia had a higher risk of poor self-rated happiness than non-volunteers with insomnia. To the best of our knowledge, this is the first study addressing how volunteer participation modulates the impact of insomnia on subjective well-being.

### Advantage of volunteer participation on self-rated health outcomes but not on HRQoL

Our findings regarding the association between insomnia and poor subjective well-being in older adults were consistent with those reported previously [8–10]. By contrast, volunteer participation showed an independent relationship with better self-rated health and happiness, which was also consistent with previous findings [47, 48]. However, it is alarming that the link between volunteers and poor HRQoL disappeared. Regarding the essential construct, self-rated health and happiness denote the final perceived global health and happiness, which are determined by positive statements [35]; however, the SF-12v2-defined HRQoL concerns negative mental and physical conditions and their impacts on daily function [37]. In a previous study from the Yilan study series, the magnitude of association between the MCS and scores of the Hospital Anxiety and Depression Scale (*r* = -0.583) was greater than that between MCS and self-rated happiness (*r* = 0.357) [35]. This finding was consistent with our argument that self-rated happiness differs from the MCS; it also emphasized the necessity to consider comprehensive instruments while examining the impact of sleep–wake related disturbance on subjective well-being.

### Differential moderation effect of volunteer participation on subjective well-being

In our findings, volunteer participation did not moderate the relationship between insomnia, self-rated health, and PCS. It is worth noting that volunteer participation per se was directly associated with better self-rated health but failed to moderate the relationship between insomnia and self-rated health. When compared with self-oriented volunteering, other-oriented volunteer participation was found to be associated with better self-rated health [49]. This finding suggests that the extent of enhancement of self-rated health may depend on the nature of one’s motivation as a volunteer. Accordingly, the adverse effects of insomnia on older adults, including daytime fatigue and poor cognition [10], may mitigate their capability of being other-oriented. As a result, volunteer participation in our study did not influence the impact of insomnia on self-rated health, as hypothesized.

We also found that volunteer participation did not associate directly with HRQoL; it indirectly influenced the relationship between insomnia and MCS. Among volunteers, insomnia was associated with a good MCS. MCS measures functional impairment secondary to negative affect, and volunteer participation correlated [21] with greater activity levels; our findings could be explained by the lower functional impairment in older adults who had insomnia but remained active in social activities.

By contrast, volunteer participation had an adverse impact on the association between insomnia and self-rated happiness. Theoretically, the construct of happiness can be divided into “hedonic happiness” and “eudaimonic happiness,” with the former emphasizing the pleasure affects arising from needs satisfaction and the latter emphasizing self-actualization as a means to gain happiness [50]. Additionally, altruism [51] and conscientiousness [52] in volunteers may contribute to the finding that while volunteer work did not benefit hedonic happiness, it may improve eudaimonic happiness [53]. However, insomnia may lead to compromised cognitive function, such as episodic memory, problem solving, and working memory [54]; this may cause more frustration to older volunteers with insomnia owing to the high cognitive function demand required in volunteering activities. Therefore, the process of cultivating eudaimonic happiness in volunteers may be hampered, leading to increased influence of insomnia on self-rated happiness.

Furthermore, stress coping mechanisms of older volunteers with insomnia may magnify its adverse impact on seeking happiness. Overdrive of attention-intention-effort pathway [29] and high personal standard concerning sleep and daytime functioning [55] features individuals with insomnia. The dual-process model of assimilative and accommodative coping suggests that well-being depends on the balanced interplay between two adaptive processes: the assimilative process and the accommodative process [56]. Older adults may intend to achieve and maintain their desired personal development by volunteer participation (the assimilative process). Insomnia is an unintended event that hinders individuals from achieving their goals owing to daytime repercussion; thus, these older adults adopt the accommodative process (or flexible goal adjustment), wherein they flexibly adjust their goals and regain balance. Compared to non-volunteers with insomnia, older volunteers with insomnia may have more prominent insomnia-related psychopathology that limits the activation of accommodative process. Further, older volunteers are more susceptible to the adverse impact of insomnia in terms of seeking happiness than non-volunteers. However, we did not collect pertinent information for the above psychological processes; thus, the actual mechanism underlying this finding needs further investigation.

### Limitations

Our study has several limitations. First, this was a cross-sectional study; thus, causal inference was not allowed. Specifically, individuals with better subjective well-being are more likely to participate in volunteering; conversely, volunteer participation also improves subjective well-being [13]. Thus, the potential bidirectional relationship between volunteering and subjective well-being hindered us from making any temporal inference. Second, medical morbidities were confirmed by the self-report of participants; therefore, their validity may have been compromised. However, the validity for dual criteria (self-reported morbidity and self-reported treatment) to establish diagnosis is confirmed in literature [57]. Therefore, the information bias on medical morbidities should be limited. Third, details of volunteer participation, such as type, frequency, and duration, were not collected. Thus, the genuine components of volunteering that have the moderation effect remain unknown. A structured instrument, such as the “Manual on the measurement of volunteer work” [58] that systemically quantifies and categorizes volunteering work is warranted to in future research. Fourth, owing to insufficient references, we arbitrarily defined the lowest tertile of each measurement as poor subjective well-being. Various cutoffs may yield different findings, but our sensitivity analyses supported the lowest tertile as the optimal cutoff. Finally, we only included older adults residing in the community; thus, the sociodemographic distribution of participants in this study may have differed from that of participants registered in Yilan city. These selection biases could limit the generalizability of our findings.

### Implications

Identifying individuals who would benefit the most from volunteer participation would assist in establishing precise interventions in health promotion programs in the community. Our findings suggested that older adults with insomnia may be one of the target populations that benefit the most (in terms of preventing negative affect) by participating in volunteering work. By contrast, regarding the positive affect, it remains unknown why volunteering amplified the impact of insomnia on self-rated happiness. In future, longitudinal studies considering volunteer characteristics and the two constructs of happiness are warranted to examine the underlying mechanisms of the relationship of insomnia and volunteer participation to subjective well-being.

## Conclusions

Our findings demonstrate the extent to which physical condition (insomnia) influences subjective well-being; they also illustrate how social factor (volunteer participation) modifies the relationship between physical conditions and subjective well-being. Although volunteer participation is not directly linked to better sleep [11], it may modify the impact of physical conditions on subjective well-being. This study also highlighted the necessity of evaluating subjective well-being by means of multidimensional instruments.

## Supplementary Information

**Additional file 1.**

## Data Availability

The datasets generated during and analyzed during the current study are not publicly available due to the risk of compromising participant confidentiality but are available from the corresponding author on reasonable request.

## Abbreviations

CI: Confidence interval
HRQoL: The health-related quality of life
MCS: The mental component summary
ORs: Odds ratios
PCS: The physical component summary
SF-12v2: The Short-Form 12 Health Survey Version 2
