# Supplementary material for: Volunteer participation differentially moderates the association between insomnia and poor subjective well-being in community-dwelling older adults: the Yilan study, Taiwan
Source: BMC Geriatr. 2022 Apr 13;22:324. doi: 10.1186/s12877-022-03004-8 (PMC9009056; doi:10.1186/s12877-022-03004-8)
Supplement: Supplementary file 1 — Additional file 1. [file 12877_2022_3004_MOESM1_ESM.docx]

| Supplement Table S1. Comparisons of sociodemographic characteristics of participants of the Yilan study and registered data in Yilan city | | | |
| --- | --- | --- | --- |
|  | The Yilan Study  (n= 3785) | Registered data in Yilan City  (n=11440) | *p*-value for χ2 of goodness-of-fit test |
|  | n (%) | n (%) |  |
| Age (years) |  |  |  |
| < 75 | 1656 (43.8) | 6193 (54.1) | χ^2^= 4.11, *p*=0.04 |
| ≥ 75 | 2129 (56.2) | 5247 (45.9) |  |
| Sex |  |  |  |
| Male | 1630 (43.1) | 5330 (46.6) | χ^2^= 0.52, *p*= 0.47 |
| Female | 2155 (56.9) | 6110 (53.4) |  |
| Education status |  |  |  |
| Literate | 3003 (79.3) | 9340 (90.3) | χ^2^= 14.58, *p<*0.001 |
| Illiterate | 782 (20.7) | 1000 (9.7) |  |

| Supplement table S2. Characteristics of sociodemographics and lifestyle of participants associated with measurements of subjective well-being (n=3785) | | | | | | | | | | | | | | | | | | | |
| --- | --- | --- | --- | --- | --- | --- | --- | --- | --- | --- | --- | --- | --- | --- | --- | --- | --- | --- | --- |
|  | Self-rated | | | | | | |  | Short Form-12 | | | | | | | | | | |
|  | Health | | |  | Happiness | | |  | Physical component summary | | | |  | | Mental component summary | | | |  |
|  | Lowest tertile | Upper two tertiles | *p*-value  for χ^2^ |  | Lowest tertile | Upper two tertiles | *p*-value  for χ^2^ |  | Lowest tertile | Upper two tertiles | *p*-value  for χ^2^ |  | | Lowest tertile | | Upper two tertiles | *p*-value  for χ^2^ |  |  |
|  | n (%) | n (%) |  |  | n (%) | n (%) |  |  | n (%) | n (%) |  |  |  | n (%) | | n (%) |  |  |  |
| Age (years) |  |  |  |  |  |  |  |  |  |  |  |  | |  | |  |  |  |  |
| < 75 | 578 (40.4) | 1078 (45.8) | 0.001 |  | 690 (38.8) | 966 (48.1) | <0.001 |  | 401 (31.6) | 1255 (49.8) | <0.001 |  | | 509 (39.8) | | 1147 (45.8) | <0.001 |  |  |
| ≥ 75 | 852 (59.6) | 1277 (54.2) |  |  | 1087 (61.2) | 1042 (51.9) |  |  | 866 (68.4) | 1263 (50.2) |  |  | | 769 (60.2) | | 1360 (54.2) |  |  |  |
| Sex |  |  |  |  |  |  |  |  |  |  |  |  | |  | |  |  |  |  |
| Female | 864 (60.4) | 1291 (54.8) | 0.001 |  | 1041 (58.6) | 1114 (55.5) | 0.054 |  | 756 (59.7) | 1399 (55.6) | 0.02 |  | | 790 (61.8) | | 1365 (54.4) | <0.001 |  |  |
| Male | 566 (39.6) | 1064 (45.2) |  |  | 736 (41.4) | 894 (44.5) |  |  | 511 (40.3) | 1119 (44.4) |  |  | | 488 (38.2) | | 1142 (45.6) |  |  |  |
| Body mass index (kg/m^2^) |  |  |  |  |  |  |  |  |  |  |  |  | |  | |  |  |  |  |
| < 18.5 | 73 (5.1) | 86 (3.7) | <0.001 |  | 89 (5.0) | 70 (3.5) | <0.001 |  | 55 (4.3) | 104 (4.1) | <0.001 |  | | 66 (5.2) | | 93 (3.7) | <0.001 |  |  |
| 18.5-23.9 | 499 (34.9) | 941 (40.0) |  |  | 667 (37.5) | 773 (38.5) |  |  | 401 (31.7) | 1039 (41.3) |  |  | | 497 (38.9) | | 943 (37.6) |  |  |  |
| > 23.9 | 740 (51.7) | 1267 (53.8) |  |  | 893 (50.3) | 1114 (55.5) |  |  | 657 (51.9) | 1350 (53.6) |  |  | | 605 (47.4) | | 1402 (55.9) |  |  |  |
| Disabled | 118 (8.3) | 60 (2.5) |  |  | 128 (7.2) | 5 (2.5) |  |  | 153 (12.1) | 25 (1.0) |  |  | | 109 (8.5) | | 69 (2.8) |  |  |  |
| Education status |  |  |  |  |  |  |  |  |  |  |  |  | |  | |  |  |  |  |
| Literate | 1047 (73.2) | 1956 (83.1) | <0.001 |  | 1296 (72.9) | 1707 (85.0) | <0.001 |  | 905 (71.4) | 2098 (83.3) | <0.001 |  | | 978 (76.5) | | 2025 (80.8) | 0.002 |  |  |
| Illiterate | 383 (26.8) | 399 (16.9) |  |  | 481 (27.1) | 301 (15.0) |  |  | 362 (28.6) | 420 (16.7) |  |  | | 300 (23.5) | | 482 (19.2) |  |  |  |
| Marital status |  |  |  |  |  |  |  |  |  |  |  |  | |  | |  |  |  |  |
| Married | 894 (62.8) | 1571 (66.9) | 0.01 |  | 1077 (60.8) | 1388 (69.3) | 0.01 |  | 761 (60.2) | 1704 (67.9) | <0.001 |  | | 795 (62.5) | | 1670 (66.7) | 0.02 |  |  |
| Single/ divorced/ separated | 30 (2.1) | 26 (1.1) |  |  | 38 (2.1) | 18 (0.9) |  |  | 27 (2.1) | 29 (1.2) |  |  | | 25 (2.0) | | 31 (1.2) |  |  |  |
| Widowed | 499 (35.1) | 753 (32.0) |  |  | 656 (37.0) | 596 (29.8) |  |  | 476 (37.7) | 776 (30.9) |  |  | | 451 (35.5) | | 801 (32.0) |  |  |  |
| Living status |  |  |  |  |  |  |  |  |  |  |  |  | |  | |  |  |  |  |
| With others | 1276 (89.3) | 2082 (88.4) | 0.42 |  | 1572 (88.5) | 1786 (89.0) | 0.64 |  | 1153 (91.1) | 2205 (87.6) | 0.001 |  | | 1133 (88.7) | | 2225 (88.8) | 0.95 |  |  |
| Alone | 153 (10.7) | 272 (11.6) |  |  | 204 (11.5) | 221 (11.0) |  |  | 112 (8.9) | 313 (12.4) |  |  | | 144 (11.3) | | 281 (11.2) |  |  |  |
| Frequency of exercise per week |  |  |  |  |  |  |  |  |  |  |  |  | |  | |  |  |  |  |
| < 3/ week | 728 (50.9) | 863 (36.7) | <0.001 |  | 893 (50.3) | 698 (34.8) | <0.001 |  | 728 (57.5) | 863 (34.3) | <0.001 |  | | 655 (51.3) | | 936 (37.4) | <0.001 |  |  |
| ≥ 3/ week | 702 (49.1) | 1491 (63.3) |  |  | 884 (49.7) | 1309 (65.2) |  |  | 539 (42.5) | 1654 (65.7) |  |  | | 623 (48.7) | | 1570 (62.6) |  |  |  |
| Volunteer |  |  |  |  |  |  |  |  |  |  |  |  | |  | |  |  |  |  |
| No | 1316 (92.4) | 2017 (86.3) | <0.001 |  | 1642 (92.9) | 1691 (84.8) | <0.001 |  | 1184 (94.0) | 2149 (85.9) | <0.001 |  | | 1149 (90.5) | | 2184 (87.7) | 0.01 |  |  |
| Yes | 108 (7.6) | 320 (13.7) |  |  | 125 (7.1) | 303 (15.2) |  |  | 76 (6.0) | 352 (14.1) |  |  | | 121 (9.5) | | 307 (12.3) |  |  |  |
| Smoking status |  |  |  |  |  |  |  |  |  |  |  |  | |  | |  |  |  |  |
| Non-smoker | 1080 (75.5) | 1786 (75.8) | 0.002 |  | 1307 (73.6) | 1559 (77.6) | 0.002 |  | 946 (74.7) | 1920 (76.3) | 0.06 |  | | 999 (78.2) | | 1867 (74.5) | 0.01 |  |  |
| Ex- smoker | 241 (16.9) | 327 (13.9) |  |  | 305 (17.2) | 263 (13.1) |  |  | 213 (16.8) | 355 (14.1) |  |  | | 187 (14.6) | | 381 (15.2) |  |  |  |
| Current smoker | 109 (7.6) | 242 (10.3) |  |  | 165 (9.3) | 186 (9.3) |  |  | 108 (8.5) | 243 (9.7) |  |  | | 92 (7.2) | | 259 (10.3) |  |  |  |
| Drinking status |  |  |  |  |  |  |  |  |  |  |  |  | |  | |  |  |  |  |
| Non-drinker | 1213 (84.8) | 1881 (79.9) | <0.001 |  | 1484 (83.5) | 1610 (80.2) | <0.001 |  | 1077 (85.0) | 2017 (80.1) | <0.001 |  | | 1101 (86.2) | | 1993 (79.5) | <0.001 |  |  |
| Ex- drinker | 79 (5.5) | 119 (5.1) |  |  | 104 (5.9) | 94 (4.7) |  |  | 80 (6.3) | 118 (4.7) |  |  | | 53 (4.1) | | 145 (5.8) |  |  |  |
| Current drinker | 138 (9.7) | 355 (15.1) |  |  | 189 (10.6) | 304 (15.1) |  |  | 110 (8.7) | 383 (15.2) |  |  | | 124 (9.7) | | 369 (14.7) |  |  |  |

| Supplement table S3. Clinical characteristics of participants associated with measurements of subjective well-being (n=3785) | | | | | | | | | | | | | | | |
| --- | --- | --- | --- | --- | --- | --- | --- | --- | --- | --- | --- | --- | --- | --- | --- |
|  | Self-rated | | | | | | |  | Short Form-12 | | | | | | |
|  | Health | | |  | Happiness | | |  | Physical component summary | | |  | Mental component summary | | |
|  | Lowest tertile | Upper two tertiles | *p*-value  for χ^2^ |  | Lowest tertile | Upper two tertiles | *p*-value  for χ^2^ |  | Lowest tertile | Upper two tertiles | *p*-value  for χ^2^ |  | Lowest tertile | Upper two tertiles | *p*-value  for χ^2^ |
|  | n (%) | n (%) |  |  | n (%) | n (%) |  |  | n (%) | n (%) |  |  | n (%) | n (%) |  |
| Medical history |  |  |  |  |  |  |  |  |  |  |  |  |  |  |  |
| Diabetes mellitus | 417 (29.2) | 471 (20.0) | <0.001 |  | 451 (25.4) | 437 (21.8) | 0.01 |  | 357 (28.2) | 531 (21.1) | <0.001 |  | 321 (25.2) | 567 (22.7) | 0.09 |
| Hypertension | 924 (64.7) | 1228 (52.2) | <0.001 |  | 1088 (61.3) | 1064 (53.1) | <0.001 |  | 811 (64.2) | 1341 (53.3) | <0.001 |  | 738 (57.9) | 1414 (56.5) | 0.41 |
| Heart disease | 594 (41.6) | 603 (25.7) | <0.001 |  | 651 (36.8) | 546 (27.2) | <0.001 |  | 651 (36.8) | 546 (27.2) | <0.001 |  | 458 (36.0) | 739 (29.5) | <0.001 |
| Hyperlipidemia | 370 (25.9) | 471 (20.1) | <0.001 |  | 421 (23.7) | 420 (21.0) | 0.04 |  | 316 (25.0) | 525 (20.9) | 0.004 |  | 292 (22.9) | 549 (22.0) | 0.51 |
| Stroke | 107 (7.5) | 87 (3.7) | <0.001 |  | 114 (6.4) | 80 (4.0) | 0.001 |  | 135 (10.7) | 59 (2.3) | <0.001 |  | 91 (7.1) | 103 (4.1) | <0.001 |
| Snore | 17 (1.2) | 26 (1.1) | 0.82 |  | 14 (0.8) | 29 (1.5) | 0.06 |  | 16 (1.3) | 27 (1.1) | 0.60 |  | 8 (0.6) | 35 (1.4) | 0.04 |
| Hospital Anxiety Depression Scale |  |  |  |  |  |  |  |  |  |  |  |  |  |  |  |
| Depression ≥ 6 | 268 (18.7) | 175 (7.4) | <0.001 |  | 326 (18.3) | 117 (5.8) | <0.001 |  | 256 (20.2) | 187 (7.4) | <0.001 |  | 311 (24.3) | 132 (5.3) | <0.001 |
| Anxiety≥ 3 | 682 (47.7) | 663 (28.2) | <0.001 |  | 835 (47.0) | 510 (25.4) | <0.001 |  | 548 (43.3) | 797 (31.7) | <0.001 |  | 735 (57.5) | 610 (24.3) | <0.001 |
| Groningen Activity Restriction Scale ≥ 19 | 667 (63.0) | 492 (31.5) | <0.001 |  | 757 (56.4) | 402 (31.5) | <0.001 |  | 813 (90.2) | 346 (20.1) | <0.001 |  | 515 (59.3) | 644 (36.8) | <0.001 |
| Athens Insomnia Scale ≥ 5 | 436 (30.5) | 370 (15.7) | <0.001 |  | 528 (29.7) | 278 (13.8) | <0.001 |  | 363 (28.7) | 443 (17.6) | <0.001 |  | 398 (31.2) | 408 (16.3) | <0.001 |
| Taking hypnotics in the past one month | 468 (32.9) | 381 (16.2) | <0.001 |  | 532 (30.1) | 317 (15.8) | <0.001 |  | 392 (31.0) | 457 (18.2) | <0.001 |  | 392 (31.0) | 457 (18.2) | <0.001 |
| Epworth Sleepiness Scale ≥ 11 | 262 (18.3) | 340 (14.4) | 0.002 |  | 315 (17.7) | 287 (14.3) | <0.001 |  | 255 (20.1) | 347 (13.8) | <0.001 |  | 257 (20.1) | 345 (13.8) | <0.001 |

| Supplement table S4. Summary of the examinations for interaction terms in different cutoffs of subjective wellbeing | | | | | | | | | |
| --- | --- | --- | --- | --- | --- | --- | --- | --- | --- |
|  | Self-rated | | | |  | Short Form-12 | | | |
|  | Health | | Happiness | |  | Physical component | | Mental component | |
|  | Adjusted OR (95% CI) | *p*-value | Adjusted OR (95% CI) | *p*-value |  | Adjusted OR (95% CI) | *p*-value | Adjusted OR (95% CI) | *p*-value |
| Median scores | ≤70 | | ≤75 | |  | ≤50.03 | | ≤61.48 | |
| n (%) | 2390 (63.1) | | 1981 (52.3) | |  | 1898 (50.1) | | 1906 (50.4) | |
| Athens Insomnia Scale x Volunteer | 1.32 (0.62-2.82) | 0.47 | 1.77 (0.86-3.65) | 0.12 |  | 1.22 (0.50-2.98) | 0.66 | 0.63 (0.31-1.29) | 0.21 |
|  |  |  |  |  |  |  |  |  |  |
| Lowest tertile scores | ≤60 | | ≤70 | |  | ≤44.36 | | ≤57.38 | |
| n (%) | 1430 (37.8) | | 1777 (46.9) | |  | 1267 (33.5) | | 1278 (33.8) | |
| Athens Insomnia Scale x Volunteer | 1.09 (0.52-2.25) | 0.83 | 2.26 (1.10-4.65) | 0.03 |  | 1.29 (0.49-3.44) | 0.61 | 0.39 (0.18-0.87) | 0.02 |
|  |  |  |  |  |  |  |  |  |  |
| Lowest quartile scores | ≤60 | | ≤60 | |  | ≤41.14 | | ≤54.62 | |
| n (%) | 1430 (37.8) | | 1011 (26.7) | |  | 948 (25.0) | | 949 (25.1) | |
| Athens Insomnia Scale x Volunteer | 1.09 (0.52-2.25) | 0.83 | 1.77 (0.80-3.94) | 0.16 |  | 0.98 (0.32-2.96) | 0.96 | 0.38 (0.16-0.91) | 0.03 |
| Covariate: age, sex, body mass index, education status, marital status, living status, frequency of exercise per week, volunteer, smoking status, drinking status, diabetes mellitus, hypertension, cardiovascular disease, hyperlipidemia, stroke, snore, Hospital Anxiety Depression Scale-depression, Hospital Anxiety Depression Scale-anxiety, Groningen Activity Restriction Scale, Athens Insomnia Scale, taking hypnotics in the past one month, and Epworth Sleepiness Scale | | | | | | | | | |
